# Substantial Generalization of Sensorimotor Learning from Bilateral to Unilateral Movement Conditions

**DOI:** 10.1371/journal.pone.0058495

**Published:** 2013-03-07

**Authors:** Jinsung Wang, Yuming Lei, Khongchee Xiong, Katie Marek

**Affiliations:** Department of Kinesiology, The University of Wisconsin, Milwaukee, Wisconsin, United States of America; The University of Western Ontario, Canada

## Abstract

Controversy exists regarding whether bimanual skill learning can generalize to unimanual performance. For example, some investigators showed that dynamic adaptation could only partially generalize between bilateral and unilateral movement conditions, while others demonstrated complete generalization of visuomotor adaptation. Here, we identified three potential factors that might have contributed to the discrepancy between the two sets of findings. In our first experiment, subjects performed reaching movements toward eight targets bilaterally with a novel force field applied to both arms, then unilaterally with the force field applied to one arm. Results showed that the dynamic adaptation generalized completely from bilateral to unilateral movements. In our second experiment, the same force field was only applied to one arm during both bilateral and unilateral movements. Results indicated complete transfer again. Finally, our subjects performed reaching movements toward a single target with the force field or a novel visuomotor rotation applied only to one arm during both bilateral and unilateral movements. The reduced breadth of experience obtained during bilateral movements resulted in incomplete transfer, which explains previous findings of limited generalization. These findings collectively suggest a substantial overlap between the neural processes underlying bilateral and unilateral movements, supporting the idea that bilateral training, often employed in stroke rehabilitation, is a valid method for improving unilateral performance. However, our findings also suggest that while the neural representations developed during bilateral training can generalize to facilitate unilateral performance, the extent of generalization may depend on the breadth of experience obtained during bilateral training.

## Introduction

Sensorimotor adaptation allows the nervous system a highly flexible control that can account for temporary, but predictable changes in response to varying constraints of a given task [1]. To understand the nature of sensorimotor adaptation, various types of generalization paradigms have been used, which include examining transfer of visuomotor or dynamic adaptation across different conditions within the same arm [2]–[4] or between the arms [5]–[7]. More recently, investigators started examining transfer of sensorimotor adaptation between bilateral and unilateral reaching conditions [8]–[11], which has major implications for stroke rehabilitation or athletic training. Bilateral arm training, for example, is used to improve motor function of the paretic arm post stroke and seems to have facilitative effects [12]–[14]. However, these claims are only valid if bilateral training can indeed generalize to unilateral performance.

Recently, the efficacy of bilateral arm training was questioned by Nozaki and colleagues, who showed that adaptation to a novel dynamic condition could generalize between bilateral and unilateral movement conditions, but only to a limited extent. Based on this finding, they suggested that only a partial overlap exists between the neural processes underlying the two types of movement. More recently, however, we demonstrated that adaptation to a novel visuomotor condition could generalize completely from bilateral to unilateral conditions [10], [11], which contradicts Nozaki et al.’s argument.

To resolve this controversy, we identified three potential factors that might have contributed to the discrepancy between the two sets of findings. The first factor involves the nature of sensorimotor tasks: Nozaki and colleagues employed a dynamic adaptation task, whereas we employed a visuomotor adaptation task. In fact, it has been previously suggested that dynamic and visuomotor adaptation may involve distinct neural mechanisms [6], [15]. The second factor we identified concerns the fact that during bilateral adaptation, perturbations were simultaneously given to both arms in our studies, but only to one arm in Nozaki et al.’s study. The last factor concerns the breadth of experience obtained during sensorimotor adaptation. Subjects in our previous studies experienced eight target directions during reaching movements, whereas those in Nozaki et al.’s study experienced a single target. It seems plausible that a greater breadth of experience obtained during initial practice may lead to the development of a more complete sensorimotor transformation; and if so, multiple-target training during bilateral adaptation may lead to greater generalization as compared to single-target training.

In the present study, we examined the effects of these three factors on the extent of generalization of dynamic adaptation from bilateral to unilateral movement conditions in a series of three experiments. We investigated the pattern of generalization from bilateral to unilateral conditions, because generalization of motor learning in this direction would be more related to rehabilitation settings (e.g., bilateral training to improve paretic arm function post stroke), and thus more interesting to rehabilitation researchers and practitioners.

## Experiment 1

This experiment served two purposes. The first, and main, purpose was to investigate generalization of dynamic adaptation from bilateral to unilateral conditions by employing an experimental paradigm used in our previous studies (i.e., a novel sensorimotor perturbation provided to both arms, reaching movements made toward multiple targets). If limited generalization were observed in this experiment, this would indicate that the discrepancy between Nozaki et al.’s findings and our previous findings was mainly due to the differences in the nature of sensorimotor tasks (i.e., dynamic vs. visuomotor). The second purpose was to determine whether the pattern of generalization from bilateral to unilateral conditions would depend on the consistency of movement directions between the arms (i.e., target directions extrinsically or intrinsically consistent between the arms).

### Methods

#### Subjects

Subjects were 24 neurologically intact young adults (12 females and 12 males, aged between 21 and 26) who were right handed. The Edinburgh Handedness Inventory was used to determine handedness. They were recruited from the university community and paid for participation. The Institutional Review Board of the University of Wisconsin – Milwaukee approved this study; and every subject signed a written informed consent prior to his/her participation. Six subjects were tested in each of four subject groups. The number of subjects per group was determined based on a power analysis we performed using the data from our previous studies that employed similar tasks and performance measures [10], [11]. No subject participated in multiple experiments.

#### Apparatus

**Figure 1:**
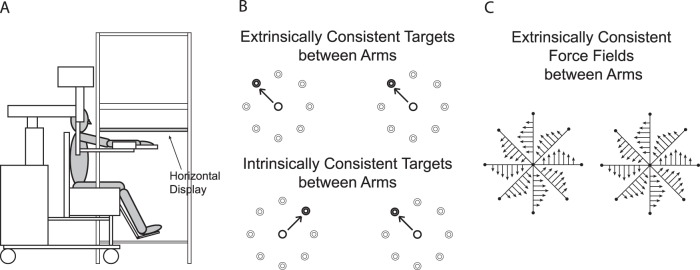
Experimental setup. A, Side view: subject sat on the KINARM chair with the arms placed under the horizontal display. B, Target was randomly displayed on one of eight target locations for each arm. Target directions were either extrinsically (top) or intrinsically (bottom) consistent between the arms during the bilateral session. C, Direction of a velocity-dependent force field applied to each arm during reaching movement. Longer arrows indicate greater forces. Force directions were always extrinsically consistent between the arms.

A bilateral robotic exoskeleton called KINARM (BKIN Technologies Ltd, Kingston, ON, Canada) was used to collect movement data. Subjects sat on the KINARM chair with their arms supported on the exoskeleton that provided gravitational support, and moved to bring their arms under a horizontal display (Fig. 1A). The KINARM was incorporated with a virtual reality system that projected visual targets on the display to make them appear in the same plane as the arms. Direct vision of the subjects’ arm was blocked; and a cursor representing the tip of their index finger was provided to guide their reaching movements. The 2-D position data of the hand, elbow and shoulder were sampled at 1,000 Hz, low pass filtered at 15 Hz, and differentiated to yield resultant velocity and acceleration values. Computer algorithms for data processing and analysis were written in MATLAB.

#### Experimental design

Prior to movement, one of eight targets (2 cm in diameter; 10 cm away from the starting position), presented in a pseudorandom sequence within each cycle (eight consecutive trials including all target directions), was displayed on the horizontal tabletop (Fig. 1B). Subjects were instructed to move their index finger from the start circle (2 cm in diameter) to the target as straight and as fast as possible in response to the appearance of the target, and stop on it. The distance between the two start circles (one for each arm) was 50 cm for all subjects, which caused the joint angles to vary across the subjects.

The experiment consisted of three sessions: baseline, bilateral and unilateral sessions. The baseline session, provided to familiarize the subjects with the general bilateral reaching task, was followed by the bilateral session during which subjects performed bilateral reaching movements toward two targets that were either extrinsically or intrinsically consistent between the arms (Fig. 1B, top and bottom, respectively). Subjects were randomly assigned to the two consistency conditions. During the subsequent unilateral session, one half of the subjects in each of the two target conditions (extrinsic, intrinsic) performed unilateral reaching movements with their left arm, and the other half with their right arm (i.e., four subject groups total, six subjects per group). The three sessions (baseline, bilateral, unilateral) consisted of 96, 192 and 96 trials, respectively, which were organized into 12 (baseline, unilateral) or 24 (bilateral) cycles. Visual feedback of the cursor representing the index finger tip was available throughout the entire experiment.

To examine adaptation to a novel dynamic condition, a velocity-dependent endpoint force field (fx, fy) was mimicked using the torque motors of the robotic exoskeleton as follows:
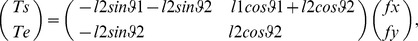



where Ts and Te are shoulder and elbow joint torques; l1 and l2 are upper arm and forearm lengths; φ1 and φ2 are shoulder and elbow angles. The force fields were fx = −15vy (−fx for left arm), fy = 15vx, where vy and vx are the y- and x-components of the endpoint velocity (m/s), respectively, and force is in Newtons. This force field was provided to both arms simultaneously during the bilateral session and to the moving arm during the unilateral session. During the bilateral session, the direction of the force fields applied to the arms was always extrinsically consistent (Fig. 1C). The force fields were only applied in the counter-clockwise direction throughout all experiments.

#### Data analysis

As our main performance measure, we calculated direction error at peak velocity (V_max_), which was calculated as the angular difference between the vectors defined by the target and by the hand-path position at movement start and at V_max_.

For statistical analysis, a repeated-measures ANOVA was conducted to examine the main effects of, and the interaction effects among, three variables: target consistency (extrinsically vs. intrinsically consistent; a between-subject factor), arm (left vs. right; a within-subject factor) and session (bilateral vs. unilateral; a within-subject factor). For the factor ‘session’, the mean direction error of the last two cycles (cycles 23 and 24) from the bilateral session and that of cycle 1 from the unilateral session were used. For the bilateral session, the mean direction error of the last two cycles was used because this mean error would reflect a more stable final adaptation level as compared to the direction error of the last cycle alone (e.g., performance in a single cycle near the end of a session could be influenced by certain factors such as boredom and fatigue). For the unilateral session, the direction error of only the first cycle was used because the performance at the very first cycle best reflects the extent of immediate transfer from the bilateral session. (Averaging the errors from cycles 1 and 2 is not ideal because a dramatic improvement typically occurs from cycle 1 to cycle 2.) The alpha level was set at.05 for statistical significance.

### Results

**Figure 2:**
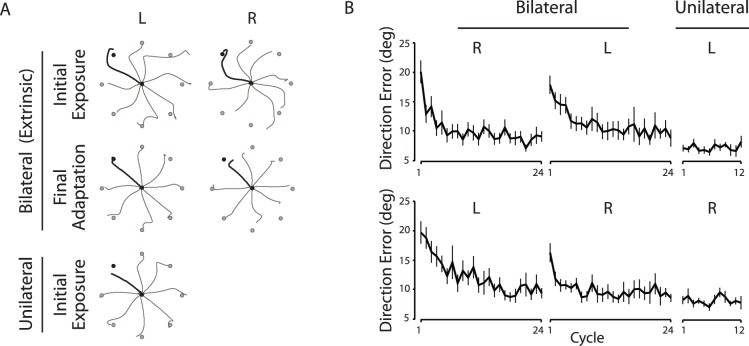
Reaching performance observed when the force field was provided to both arms during the bilateral session. A, Hand-paths obtained from a representative subject who reached toward extrinsically consistent targets during the bilateral session and used the left arm during the unilateral session. Hand-paths represent the first (rows 1, 3) or the last (row 2) eight consecutive trials in each session. Pairs (L and R) of black lines in the bilateral session indicate target directions presented simultaneously for bilateral performance. L above the hand-paths indicates left arm, R right arm. B, Mean direction errors. Every data point shown on X axis represents the average of 8 consecutive trials (cycle) across subjects (mean ± SE). Direction errors for the subjects who performed the unilateral task with the left (top) or the right (bottom) arm are shown separately (data collapsed across two target-consistency conditions due to lack of significant differences).

Figure 2A illustrates typical hand-paths from a representative subject in one of the four subject groups (those who experienced the extrinsically consistent targets during the bilateral session and used the left arm during the unilateral session). Hand-paths shown in rows 1 and 2 represent the first and the last eight consecutive trials during the bilateral session, respectively; and those in row 3 represent the first eight consecutive trials during the unilateral session. Upon initial exposure to the force field during the bilateral session (row 1), the hand-paths obtained at the first cycle of both left and right arm performances were deviated substantially from a straight line between the start circle and the target. Following adaptation to the force field, these paths became relatively straight and substantially more accurate (row 2). The hand-paths obtained during the unilateral session were relatively straight from the first cycle, indicating largely facilitative effects of bilateral training on subsequent unilateral performance. The facilitative effects of bilateral training, indicated by relatively straight and accurate hand-paths at the first cycle of the unilateral session, appeared to be similar across the four subject groups.

Figure 2B illustrates the mean values (± SE) of direction error for two conditions: one in which the subjects performed the unilateral task with the left arm (top) and the other in which they performed the same task with the right arm (bottom). The repeated-measures ANOVA showed that none of the three factors (target consistency, arm, session) had a significant main effect; and no interaction effect among those factors was significant. (Data shown in Figure 2B were collapsed across the two target-consistency conditions because no significant differences were observed between them.)

Additional post hoc analyses indicated that peak tangential velocity during reaching movements was not statistically different between bilateral and unilateral performances at the first and the last cycles.

## Experiment 2

The results from experiment 1 indicated substantial generalization from the bilateral to unilateral sessions when the force field was provided to both arms. The purpose of experiment 2 was to investigate the generalization pattern from a bilateral to a unilateral session when the force field was only applied to one arm in both sessions.

### Methods

#### Subjects

Subjects were 5 neurologically intact young adults (3 females and 2 males, aged between 21 and 25) who were right handed. No subject participated in the other experiments.

#### Experimental design

This experiment employed the same reaching tasks described above, and also consisted of the three sessions (baseline, bilateral, unilateral). During the bilateral session, however, all subjects experienced extrinsically consistent target directions between the arms. Extrinsically consistent target directions were used, because moving the two arms in extrinsically consistent directions was thought to be rather unnatural as compared with moving them in intrinsically consistent directions [16]–[19]; and we reasoned that if substantial generalization were observed in this condition, a good amount of generalization would be observed in the other (more natural) condition as well. During the unilateral session, all subjects performed the reaching task with their left arm. The left arm was tested, because the subjects in Nozaki et al.’s study also used the left arm during the unilateral session. The force field was only provided to the left arm during both the bilateral and unilateral sessions. The three sessions (baseline, bilateral, unilateral) consisted of 96, 192 and 48 trials (i.e., 12, 24 and 6 cycles), respectively.

#### Data analysis

For statistical analysis, direction errors from the aforementioned condition (i.e., the force field applied only to the left arm) were compared with those from one of the four conditions included in experiment 1 (i.e., the force field applied to both arms, target directions extrinsically consistent between the arms, the left arm tested during the unilateral session). A repeated-measures ANOVA was conducted to examine the main effects of, and the interaction effect between, two variables: force field (applied to both arms vs. one arm; a between-subject factor) and session (bilateral vs. unilateral; a within-subject factor). For the factor ‘session’, the mean direction error of the last two cycles (cycles 23 and 24) from the bilateral session and that of cycle 1 from the unilateral session were used. The alpha level was set at.05 for statistical significance.

### Results

**Figure 3:**
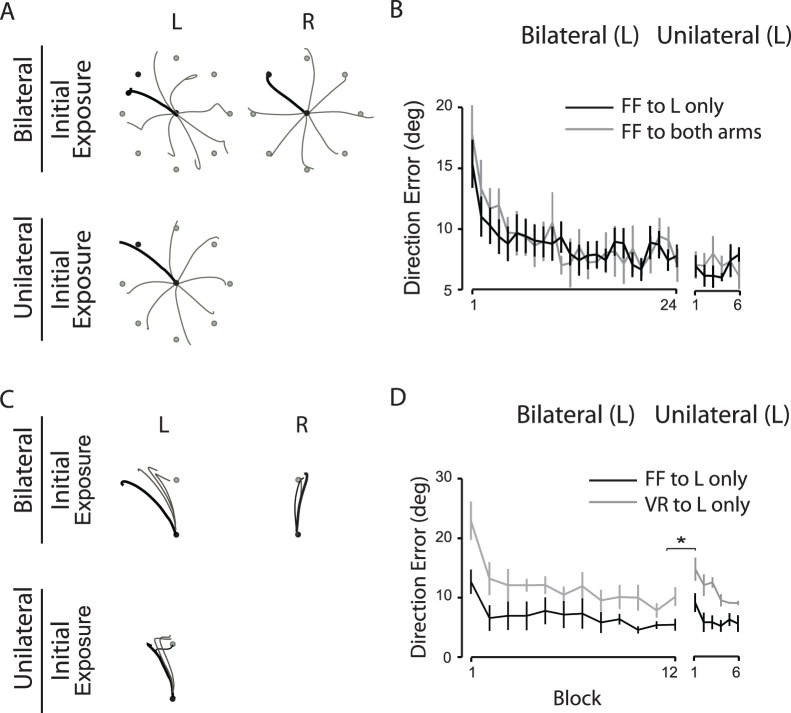
Reaching performance observed when the sensorimotor perturbation was only provided to the left arm. A, Hand-paths from a representative subject who experienced the force field provided only to the left arm during both the bilateral and unilateral sessions. B, Mean direction errors. Every data point shown on X axis represents the average of 8 consecutive trials (cycle) across subjects (mean ± SE) when the force field was provided only to the left arm (FF to L only, black lines) or to both arms (FF to both arms, grey lines). C, Hand-paths from a representative subject who experienced the force field provided only to the left arm during both the bilateral and unilateral sessions and reached toward a single target. D, Mean direction errors. Every data point shown on X axis represents the average of 8 consecutive trials (block) across subjects (mean ± SE) in the dynamic task condition (FF to L only, black lines) or in the visuomotor task condition (VR to L only, grey lines). * P<.05.

As observed in experiment 1, the hand-paths of a representative subject at the first cycle of the unilateral session (Fig. 3A, bottom) were substantially straighter than those observed at the first cycle of the bilateral session (top, left). The left arm performance, indicated by the mean direction errors (+ SE), improved substantially throughout the bilateral session in which the force field was provided only to the left arm (Fig. 3B, black s); and the level of performance observed at the end of the bilateral session appeared similar to that observed at the beginning of the subsequent unilateral session. The pattern of adaptation, as well as generalization, observed in this condition was similar to that observed in the other condition in which the force field was provided to both arms during the bilateral session (Fig. 3B, grey lines). The repeated-measures ANOVA indicated that neither force field nor session had a significant main effect; and the interaction effect between the two factors was not significant either, indicating substantial transfer of dynamic adaptation again.

## Experiment 3

The results from experiments 1 and 2 indicated substantial generalization from the bilateral to unilateral sessions regardless of whether the force field was provided to both arms or only to one arm. The purpose of experiment 3 was to investigate the generalization pattern from a bilateral to a unilateral session when subjects reached only toward a single target in both sessions. In this experiment, we also included a comparable condition in which subjects adapted to a novel visuomotor rotation to determine the similarities between the pattern of generalization following dynamic and visuomotor adaptation.

### Methods

#### Subjects

Subjects were 10 neurologically intact young adults (5 females and 5 males, aged between 22 and 25) who were right handed. No subject participated in the other experiments. Five subjects were tested in each subject group.

#### Experimental design

Two experimental conditions were employed: one in which the subjects adapted to a novel force field condition, and the other in which they adapted to a novel visuomotor rotation. The former condition was identical to the condition tested in experiment 2, except that the subjects only reached toward a single target (“12 o’clock” direction) with each arm during both the bilateral and unilateral sessions. In the latter condition, the cursor representing the index finger tip location was rotated 30 degrees counterclockwise about the start circle as it moved toward the same target during both the bilateral and unilateral sessions. A similar visuomotor adaptation task (i.e., 30 degree counterclockwise rotation, but reaching toward eight different targets) was employed in our previous studies [10], [11]. The three sessions (baseline, bilateral, unilateral) consisted of 48, 96 and 48 trials (6, 12 and 6 blocks, with each block representing the mean of eight consecutive trials), respectively.

#### Data analysis

A repeated-measures ANOVA was conducted to examine the main effects of, and the interaction effect between, two variables: task (dynamic vs. visuomotor; a between-subject factor) and session (bilateral vs. unilateral; a within-subject factor). For the factor ‘session’, the mean direction error of the last two cycles (blocks 11 and 12) from the bilateral session and that of block 1 from the unilateral session were used. The alpha level was set at.05 for statistical significance.

### Results

Figure 3C illustrates the hand-paths of a representative subject from the dynamic adaptation condition. The hand-paths at the first four trials of the unilateral session (Fig. 3C, bottom) were straighter than those observed at the first four trials of the bilateral session (top, left), although they still deviated substantially from a straight line between the start circle and the target. The hand-paths of the subjects tested in the visuomotor adaptation condition were almost the same as those described above, except that the deviations from a straight line between the start circle and the target were somewhat greater in the visuomotor condition.

The left arm performance, indicated by the mean direction errors (+ SE), improved substantially throughout the bilateral session in both task conditions (Fig. 3D). However, the level of performance observed at the beginning of the unilateral session was poorer than that observed at the end of the bilateral session regardless of the task condition. The repeated-measures ANOVA indicated that the task main effect was not significant, but the session main effect was (p = .002). The interaction effect between the two factors was not significant. This indicates limited generalization of both dynamic and visuomotor adaptation from the bilateral to the unilateral session.

## Discussion

We previously demonstrated that visuomotor adaptation could generalize completely from bilateral to unilateral movement conditions, regardless of the consistency between the arms in terms of target directions or visual rotation directions [10], [11]. In the present study, we demonstrated that dynamic adaptation could also generalize substantially from bilateral to unilateral conditions. Complete generalization was observed regardless of whether the force field was provided to both arms (experiment 1) or only to one arm (experiment 2). These findings indicate that the learning that occurred in the two bilateral conditions had similar effects, which is in line with the finding that learning of a force field in one arm was the same regardless of whether the other arm made movements in a null field or in a force field [20]. Our findings also indicate that the effect of bilateral training on subsequent unilateral performance is quite robust and not overly sensitive to the context of bilateral training. It has been suggested that bilateral movements in different contexts may engage distinct representations of the limb dynamics and kinematics [21]. Considering that, bilateral movements performed in our two conditions in experiment 1, one in which the movement directions were the same between the arms and the other in which the directions were opposite, might have engaged two distinct neural representations. Our results, however, were similar (statistically not different) between the two conditions, indicating that substantial generalization can occur from bilateral to unilateral conditions regardless of the contexts of bilateral training. These findings are in agreement with a recent finding that adapting to a novel disturbance torque during a bimanual tracking task facilitated performing the same task unilaterally regardless of whether the torque was applied symmetrically or asymmetrically to the two arms [22].

In experiment 3, we observed limited generalization of dynamic adaptation from the bilateral to the unilateral session when the subjects reached only toward a single target during initial training. The extent of transfer observed in this experiment is comparable to that observed in Nozaki et al.’s study (∼60% transfer observed in their study, Fig. 1f). The mean (± SE) direction errors at block 1 and blocks 11∼12 of the bilateral session and at block 1 of the unilateral session in experiment 3 were 12. 38 (±4.78), 5.45 (±1.59) and 8.53 (±2.95) degrees, respectively. This indicates that approximately 56% transfer occurred from the bilateral to the unilateral session when the subjects only experienced a single target during reaching movements. It should be noted here that the number of trials in the bilateral session was smaller in experiment 3 as compared with the number of trials in the same session in experiments 1 and 2 (96 vs 192 trials, respectively). We used a smaller number of trials because reaching toward a single target for too many trials could have a negative effect on the initial adaptation due to certain factors such as the loss of motivation and boredom. One may argue that the limited generalization observed in this experiment was influenced by the reduced number of trials. However, the number of trials for the given target direction (12 o’clock direction) was four times more in this experiment than in experiments 1 and 2 (96 vs. 24 trials, respectively). In addition, the learning curve observed in experiment 3 was very similar to that observed in the other experiments. Thus, it is unlikely that the smaller number of trials in this experiment had a substantial influence on the observed extent of generalization.

We also investigated generalization of visuomotor adaptation from the bilateral to the unilateral session in experiment 3. Our results showed limited generalization, indicated by a significant difference between the direction errors at the last blocks of the bilateral performance and those at the first block of the unilateral performance, when the subjects only experienced a single target direction. This finding is very different from the findings from our previous studies [10], [11], in which complete generalization of visuomotor adaptation occurred from the bilateral to the unilateral session when the subjects experienced eight different target directions (as our subjects did in the current study, experiment 1). This is in line with the well-known variability of practice principle of motor learning, which posits that experiences with task variations are crucial to the development of motor memories that are responsible for motor control and learning [23], [24]. It is, thus, speculated that experiencing a broader range of movement (target directions in our case) during bilateral adaptation may lead to the development of a neural representation associated with a novel sensorimotor transform (whether it is visuomotor or dynamic in nature) that is less task-specific or less context-dependent, thus resulting in greater generalization across different types of movements. This idea is also in agreement with the finding reported by Seidler [25] that experiencing a variety of motor learning paradigms can facilitate the acquisition of general, transferable knowledge about skill learning processes, regardless of similarities among the experienced paradigms (e.g., visual rotations, gain change, sequence learning).

Our current findings have an implication for rehabilitation. Whether generalization of sensorimotor adaptation investigated in our study is indeed a good model of what happens during stroke rehabilitation is an open question. Nonetheless, our findings clearly indicate that the neural mechanisms underlying this type of motor learning overlap substantially between bilateral and unilateral training, which provides support to the idea that bilateral arm training employed in stroke rehabilitation can facilitate functional recovery of the paretic arm in stroke patients with hemiparesis [12]–[14]. In stroke rehabilitation, different types of bilateral arm training are employed, such as bilateral isokinematic training, machine-assisted bilateral training, bilateral mirror therapy and bilateral priming (see [13] for review). When investigating the effects of these training methods, investigators typically employ symmetrical, as compared with asymmetrical, movements between the arms because symmetrical movements may involve the generation of similar neural commands to control the two arms, which is thought to be more beneficial for improving motor function of the paretic arm. Studies that investigated bilateral coordination in healthy young adults suggest that bilateral coordination is more stable, in terms of both temporal and spatial domains, when the arms perform symmetrical, as compared to asymmetrical, movements (e.g., [16]–[19]). Symmetrical bilateral movements have also been shown to involve similar neural processes in both hemispheres (e.g., [26]–[28]). However, our current findings, along with our previous findings [11], indicate that complete generalization of both dynamic and visuomotor learning can occur regardless of whether the arms move symmetrically or asymmetrically. A small number of investigators employed both symmetrical and asymmetrical bilateral arm training in their stroke intervention studies. However, a systematic comparison between the two types of training was not done in those studies because they had the same stroke patients experience both types of movement during bilateral training (e.g., [29]) and/or because they did not have a sufficient number of patients in the two movement conditions (e.g., [30]). Further research is necessary to determine whether symmetrical and asymmetrical bilateral training are equally effective for improving motor function of the paretic arm post stroke as well.
